# Smartphone-Based Janus Micromotors Strategy for Motion-Based Detection of Glutathione

**DOI:** 10.1021/acs.analchem.1c02947

**Published:** 2021-11-22

**Authors:** Kaisong Yuan, Carmen Cuntín-Abal, Beatriz Jurado-Sánchez, Alberto Escarpa

**Affiliations:** † Department of Analytical Chemistry, Physical Chemistry and Chemical Engineering, University of Alcala, Alcala de Henares, E-28871 Madrid, Spain; ‡ Shantou University Medical College, No. 22, Xinling Road, Shantou 515041, China; § Chemical Research Institute “Andrés M. del Río”, University of Alcala, Alcala de Henares, E-28871 Madrid, Spain

## Abstract

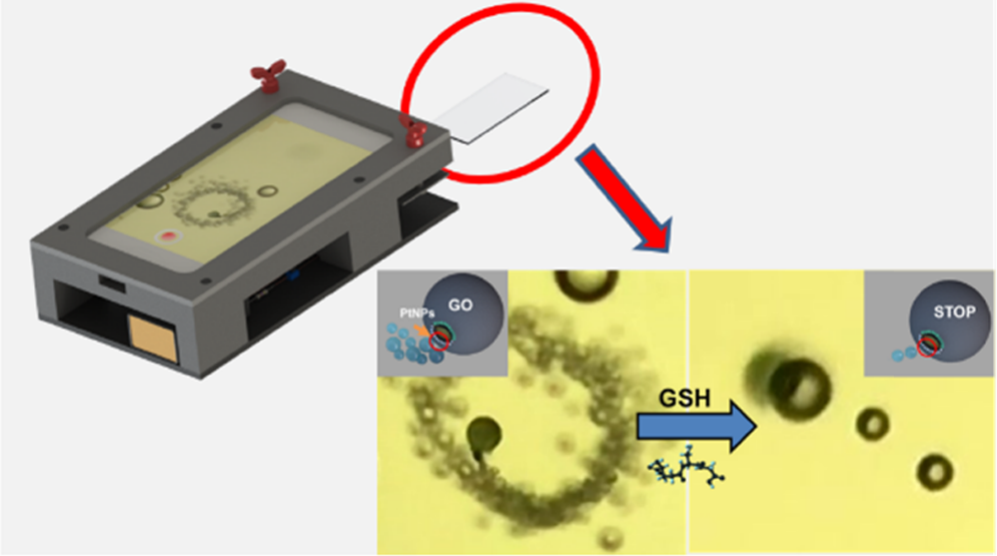

Herein, we describe a Janus micromotor smartphone platform for the motion-based detection of glutathione. The system compromises a universal three-dimensional (3D)-printed platform to hold a commercial smartphone, which is equipped with an external magnification optical lens (20–400×) directly attached to the camera, an adjustable sample holder to accommodate a glass slide, and a light-emitting diode (LED) source. The presence of glutathione in peroxide-rich sample media results in the decrease in the speed of 20 μm graphene-wrapped/PtNPs Janus micromotors due to poisoning of the catalytic layer by a thiol bond formation. The speed can be correlated with the concentration of glutathione, achieving a limit of detection of 0.90 μM, with percent recoveries and excellent selectivity under the presence of interfering amino acids and proteins. Naked-eye visualization of the speed decrease allows for the design of a test strip for fast glutathione detection (30 s), avoiding previous amplification strategies or sample preparation steps. The concept can be extended to other micromotor approaches relying on fluorescence or colorimetric detection for future multiplexed schemes.

## Introduction

Fast biomarker detection is essential for personalized healthcare and rapid disease treatment. Advances in nanofabrication allow for the miniaturization of biosensors for such purposes, which along with smartphone technology led to novel point-of-care (POCs) applications. Such miniaturized devices are easy to use, holding considerable promise to reduce costs, increase sampling throughput, and allow their use in resource-limited settings.^1−3^ In such devices, smartphones are used as detection/readout parts; thus, the integration of sample preparation compartments or reaction chambers is necessary. For example, lateral-based flow strips can be easily coupled with mobile phones for fluorescence detection of peptides indicative of heart failure^4^ or colorimetric detection of uric acid in whole blood.^2^ A paper-based plasma separation module has been used in connection with a tailor-made reservoir for colorimetric detection of total and direct bilirubin in the blood.^5^ More sophisticated designs rely on the integration of microchips for polymerase chain reaction for DNA amplification^6−8^ or to promote microbead aggregation via protein-specific linkage for prostate-specific antigen detection.^9^

Self-propelled micromotors are microscale devices capable of autonomous movement in solution.^10−15^ In the analytical field, catalytic micromotors propelled by peroxide decomposition in inner catalytic layers are the most used to date. Indeed, their autonomous motion along with versatile functionalization approaches leads to novel developments in the analytical field to perform a myriad of assays directly using ultralow (nL−μL) sample volumes.^16−20^ The high towing force, small micromotor size, and the possibility to introduce magnetic parts in its structure allow for efficient navigation and control in microfluidic chips for future smartphone-based POCs.^21,22^ A more promising approach to achieve this goal is the integration of motion-based sensing approaches based on micromotors. Wang’s group pioneer the concept after observing the enhanced movement experienced by Au–Pt nanowires in the presence of Ag^+^ ions.^23^ Changes in the speed were related to Ag^+^ concentration to develop the sensing strategy, which was later extended for DNA sensing.^24^ The limitations of the movement of nanowires in salt-rich environments led to the exploration of bubble-propelled catalytic micromotors. For example, polymer–Au micromotors with an inner catalase layer experienced diminished motion in the presence of certain ions (Hg)^25^ or nerve agents vapor plumes^26^ due to poison of the enzyme. Micromotors with inner Pt catalytic layers can also be poisoned by thiol-containing metabolites, resulting in a similar speed decrease.^27^ Antibody-modified gold-nanoparticle-polyaniline/Pt micromotors have been used in connection with secondary antibody-modified microspheres for anticarcinoembryonic antigen detection. The presence of high concentrations of the target analyte results in the aggregation of more microspheres, resulting in a speed decrease in a concentration-dependent manner.^28^ Turn-off motion approaches based on poly(3,4-ethylenedioxythiophene)/Au micromotors^29^ or Au/Ag/Ni/Au shells^30^ have been described for DNA sensing. The principle relies on DNA competition toward catalase-labeled secondary probes (responsible for the propulsion) that are released, resulting in a speed decrease. Yet, the above-mentioned approaches required the use of high-performance optical microscopes, which prevents its use in routine laboratories or in portable detection schemes.

The recent introduction of 3D printing and fast progress in smartphones allow for the integration of motion-based micromotors assays for POC diagnosis. Shafiee et al. developed a motion-based cellphone detection system for HIV-1. The systems compromise a microchip and DNA-modified micromotors consisting of platinum- and gold-modified polystyrene beads (6 μm). The detection principle consisted of (1) sample application in the reservoir of the microchip for loop-mediated isothermal amplification of the nucleic acid of HIV-1; (2) mixing of resulting amplicons with the DNA-modified micromotors; and (3) detection of the motion of the resulting assemblies in peroxide solutions. The presence of HIV-1 generates large amplicons that reduce the motion/speed of motors (turn-off). Such a decrease is used as the analytical signal for HIV detection at a concentration as low as 1000 virus particles mL^–1^ with high specificity within an hour.^31^ The same design but in a turn-on configuration was also applied for Zika virus detection. In this case, 3 μm beads modified with anti-Zika virus monoclonal antibody (anti-ZIKV mAb) are used to capture the Zika virus, followed by attachment of anti-ZIKV mAb platinum nanoparticles. The presence of virus in testing samples results in the accumulation of an increased concentration of Pt beads, causing the motion in peroxide solutions for the detection of a concentration as low as 1 particle μL^–1^.^32^ Despite their simplicity, such systems still require the use of microchips for sample pretreatment and sophisticated detection algorithms for motion detection as visual detection is not possible.

**Figure 1:**
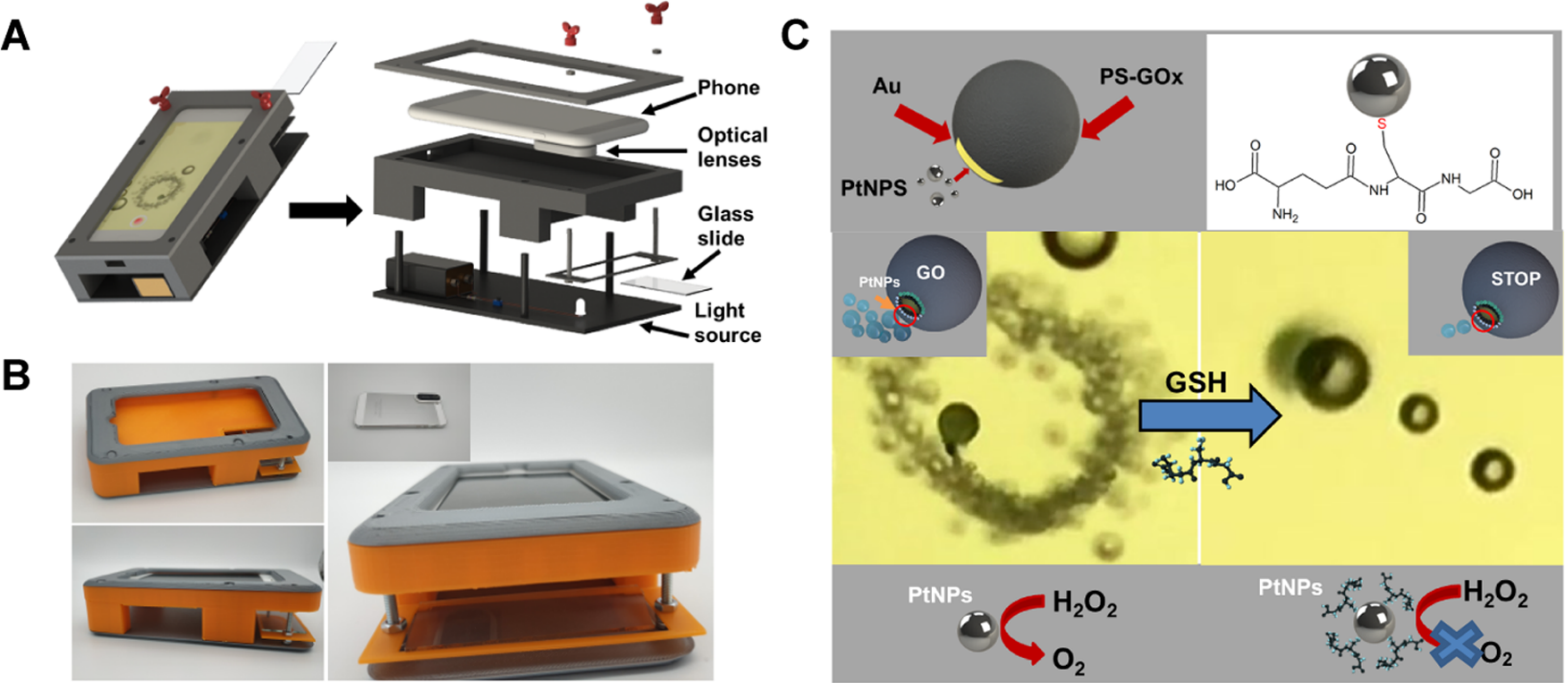
Smartphone-based platform design and proof-of-concept application for glutathione (GSH) detection. (A) Integration of the smartphone into a 3D-printed platform and description of the different parts of the device. The schematic of the assembly is described in Video S1. (B) The corresponding picture of the device. (C) Schematic of GSH detection with GO-wrapped/PtNP Janus micromotors based on the decrease in the initial speed due to inactivation of the catalytically active PtNPs by specific interactions with the thiol group present in the GSH molecule. The time-lapse microscopy images (taken from Video S2) illustrate the Janus micromotor motion in the absence (left) and presence of 160 μM of GSH (right) using the smartphone-based platform.

Inspired by previous micromotors works, herein we describe a smartphone-based detection platform for motion-based detection of relevant analytes. The system relies on the direct coupling of a high-performance commercial optical scope with the smartphone camera, allowing for the real-time observation of 20 μm graphene (GO)-wrapped/PtNPs Janus micromotor motion (see Figure 1A). A specifically designed 3D attachment allows for the integration of a light-emitting diode (LED) source for illumination, a glass slide holder to place a few microliters of the sample, and an easy system for fast focus. The main aim is to simplify even more the configuration, leading to a universal platform that can be coupled with any mobile phone. The concept is demonstrated here to detect glutathione (GSH), an important peptide biomarker that plays a critical role in cellular functions. In addition, abnormally high levels of GSH (higher than 15 mM) can be related to many diseases, such as diabetes, or viral infections, whereas extremely low levels have been associated with Alzheimer’s disease.^33−36^ Such compounds contain also a thiol group, which can attach to the PtNPs responsible for micromotor motion, using the decrease in the speed as an analytical signal. Direct visualization of the speed decrease allows for the design of a test strip for GSH detection, avoiding previous amplification strategies or sample preparation steps. The smartphone device proposed here is affordable, is user-friendly, and does not require special equipment, allowing for fast control, i.e., in epidemics and remote areas.^37^ The versatility of the strategy allows for easy integration of other micromotor-based sensing strategies relying on fluorescent or colorimetric detection of a myriad of biomarkers, allowing for the design of multiplexed schemes.

## Experimental Section

### Smartphone Device

The 3D platform for the cellphone setup was designed using SolidWorks 2015 software. The platform contains a LED source and a sample holder to place a glass slide. The 20× to 400× Universal Tip scope was purchased from Amazon and directly attached to an iPhone (Apple), which was used in all experiments. Micromotor motion was directly observed in the camera application of the phone, which was also used to record the Videos at 30 FPS. Micromotor motion was tracked with the Particle Tracker module of ImageJ free software (https://imagej.net/Particle_Tracker). Additionally, the commercial Nikon NIS Elements AR 3.2 software and tracking module was used to check the accuracy of the free software.

An inverted optical microscope (Nikon Eclipse Instrument Inc., TiS/L100) and a Zyla cMOS digital camera were used to validate the smartphone platform. Movies were captured and the speed of the micromotors tracked for comparison.

### Micromotor Synthesis

All reagents were obtained from Sigma-Aldrich (Spain) and used without further purification. Polystyrene microparticles (cat. 87896) were dropped on a clean-glass slide to generate a monolayer, which was covered with a ∼50 nm gold by sputter coating. The modified beads were released in ultrapure water by sonication (0.9 mL) and mixed with 0.1 mL of sulfhydryl-modified graphene oxide (HS-GO) (cat. 763705) for 2 h to promote attachment to gold by a thiol bond.^38^ Next, the solution was filtered to remove the excess reagents and dispersed in ultrapure water (1 mL). The PtNPs were grown in situ in the microparticles by mixing the solution with 200 μL of chloroplatinic acid hydrate (1 mg mL^–1^, cat. 398322) and 20 μL of hydrazine solution (35 wt % in H_2_O, cat. 309400) for 2 h. After that, the reaction solution was filtered with a cyclopore track-etched membrane (5 μm) to remove free PtNPs. The volume of the solution was adjusted to 1 mL with ultrapure water and remained stable for 2 months without any change in its properties.

### GSH Detection

For detection, 1 μL of micromotor solution was mixed with 1 μL of sodium dodecyl sulfate (cat. 71725, final concentration 3%), 1 μL of variable concentrations of GSH (cat. PHR1359, from 0 to 160 μM) and 1 μL of H_2_O_2_ (cat. 216763, final concentration, 5%). Videos were recorded after 30 s peroxide addition and the speed tracked as previously specified. The analytical performance was evaluated through the limit of detection (LOD), the limit of quantification (LOQ), selectivity, and recovery. Calibration plots were obtained (*n* = 5) and the LOD or LOQ were calculated as 3 or 10 times the standard deviation of the ordinate divided by the slope of the calibration linear fit. Selectivity was evaluated against 160 μM of potentially interfering species including cysteine (cat. W326305), serine (cat. S4500), leucine (cat. L8000), arginine (cat. A5006), and bovine serum albumin (cat. 05470). Recoveries were calculated by a 100-fold dilution of human serum (cat. H4522). Prior to adding the desired amount of GSH, its concentration in serum was determined with the micromotors using a microscope and a phone. Next, the samples were fortified with 3, 25, and 160 μM of GSH.

## Results and Discussion

**Figure 2:**
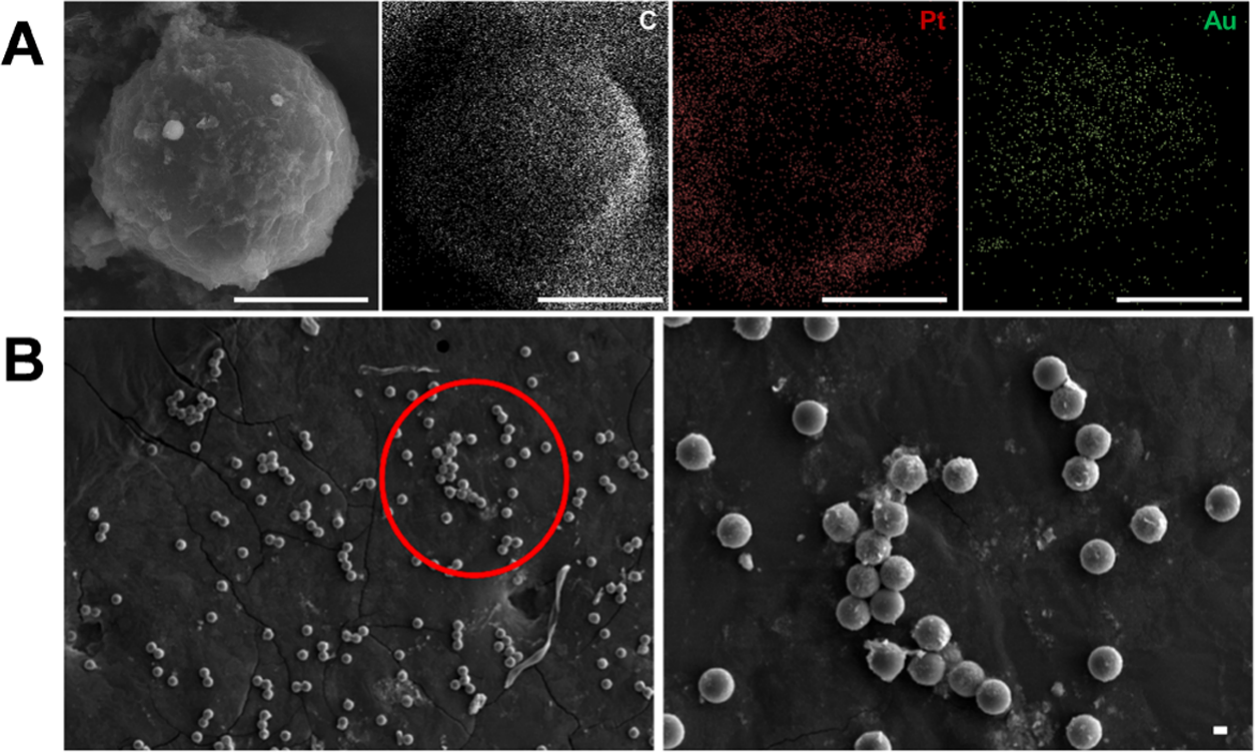
GO/PtNP Janus micromotor characterization. (A) Scanning electron microscopy (SEM) and energy-dispersive X-ray mapping (EDX) mapping showing the Janus morphology and elements distribution. (B) SEM images showing the distribution and uniform micromotor size of a drop taken from a micromotor batch. Scale bars, 10 μm.

Figure 1A illustrates the setup of the micromotor-based detection platform. It consists of a 3D-printed platform that can be easily tailored for many smartphone models and a commercial magnification lens attached to the camera of the phone (overall cost 19€, with an estimated 2-year lifetime). An animation of the assembly is shown in Video S1. As can be seen, a sample holder for the inclusion of a glass slide is incorporated, which along with easy-to-use adjustable screws allows the movement for sample focus, like a high-performance optical microscope. In that way, a real-time micromotor motion observation can be achieved, even by nonspecialized personnel. The platform was tested to develop a protocol for GSH detection in clinical samples. To this end, we use Janus micromotors whose size is ideal for observation with the designed system. Micromotors were synthesized using a strategy previously developed by our research group.^38^ Briefly, gold-sputtered PS microspheres were coated with GO modified with −SH to promote attachment via a thiol bond with an Au layer. The amount of GO for incubation with the PS-Au particles was judiciously controlled to leave uncoated a small, asymmetric Au patch. In this way, preferential growth of the catalytic Pt nanoparticles is promoted, imparting the micromotors with the asymmetric Janus character for efficient propulsion in peroxide solutions. To check this, we incubate the PS-Au particles with variable amounts of GO-SH, followed by PtNP attachment and subsequent EDX characterization. Additionally, motion propulsion experiments were tested. Under nonoptimized conditions, no motion performance is observed because GO covers the whole Au layer, preventing preferential PtNP growth and thus movement.^38^Figure 2A shows the SEM and EDX images of the Janus morphology and the elemental distribution of our micromotors. Also, as can be seen in Figure 2B, the micromotor size is uniform, with distinct Janus parts and a good density of micromotors in the same batch. While Janus micromotors were used here to demonstrate real-time detection, tubular designs or other configurations can be easily observed with our device for future motion-based micromotor sensing approaches.

The schematic of the turn-off GSH sensing is depicted in Figure 1C. Initially, micromotors propelled at a speed of over 162 ± 5 μm s^–1^ in 5% peroxide solutions. Yet, under the presence of GSH, the thiol group present in the molecule can poison the PtNPs catalyst by the specific union through a thiol bond. This blocks the catalytic active area, preventing the decomposition of peroxide by the catalyst and resulting in a decrease in the speed, which can be related to the GSH concentration. Even more, 160 μM GSH concentrations result in the almost complete stopping of the micromotor movement because all active catalytic area is blocked by such an analyte.^27^ This is further illustrated in the time-lapse microscopy images of the inset in Figure 1B and the related Video S2. The images and videos were taken using a microscope (for further details, see the Experimental Section), which clearly illustrates the micromotor movement by the naked eye and the clear differences in the movement.

**Figure 3:**
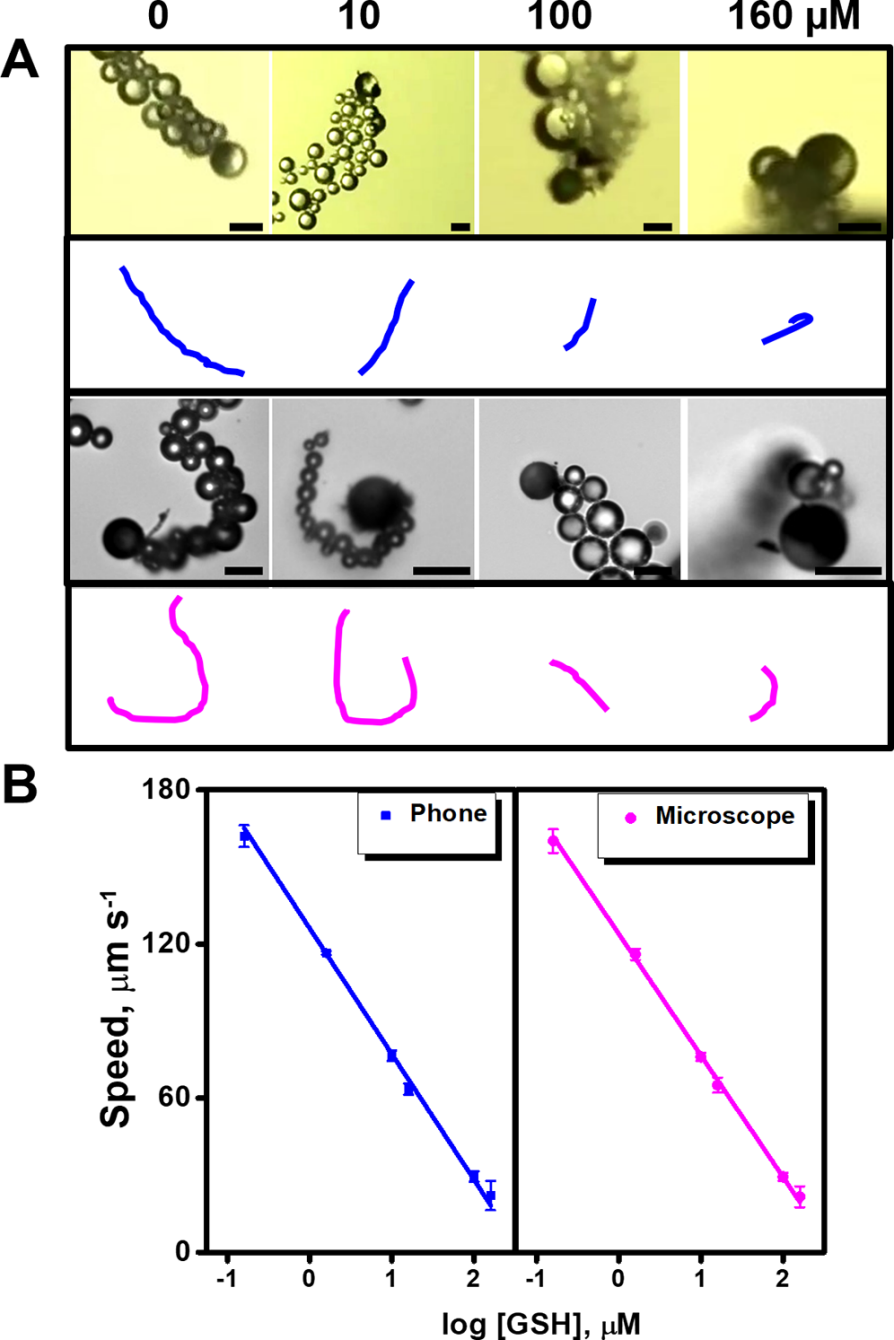
Detection strategy and calibration using a high-performance optical microscope and a smartphone. (A) Time-lapse images (taken from Video S3) and tracking lines of micromotor navigation in solutions containing increasing concentrations (0, 10, 100, and 160 μM) of GSH. The top part shows the images obtained with a smartphone and the bottom part shows the one obtained with a high-resolution optical microscope. (B) The corresponding calibration plots. Scale bars, 20 μm.

The strategy was next validated, and the results were compared to those obtained with a high-performance optical microscope. Figure 3 and related Video S3 illustrate the micromotor movement in solutions containing increasing concentrations of GSH (from 10 to 160 μM). As can be seen, as the GSH concentration increases, there is a decrease in the micromotor speed because less catalytic area is available for peroxide decomposition. This is also revealed by the bubble tail, with bigger and less abundant oxygen bubbles as GSH concentration increases. The data were processed to obtain calibration plots using the micromotor speed as an analytical signal. Figure 3B shows the calibration plots obtained using both the smartphone and the conventional optical microscope. The relevant analytic characteristics are summarized in Table 1.

**Table 1:** Analytical Characteristics of GSH Detection Using the Smartphone and the Optical Microscope

approach	linear range (μM)	*R*^2^	intercept (μm s^–1^)	slope (μm s^–1^ μM^–1^)	LOD (μM)	LOQ (μM)
microscope	2.8–160	0.997	123.9 ± 1.0	–47.4 ± 0.7	0.86	2.8
phone	2.8–160	0.998	126.1 ± 0.8	–48.0 ± 1.0	0.89	2.9

As can be seen in Table 1, similar results were obtained using both a smartphone and an optical microscope. The LODs were 0.89 and 0.86 μM for the smartphone and a microscope, respectively. The linear range span to 160 μM in both cases. Such LOD is similar to that obtained with a chemiluminescent method using MnO_2_-nanosheet-modified upconversion nanoparticles (0.9 μM)^39^ or even lower than that obtained with fluorescent approaches using polydopamine-doped nanoparticles in the presence of MnO_2_ (1.5 μM)^40^ or AuNC@BSA–MnO_2_ nanoparticles (20 μM).^41^ The LOD obtained with our method is slightly higher than that obtained with a colorimetric approach using a Ru(bpy)_3_^2+^-modified metal–organic framework in connection with the substrate 3,3′,5,5′-tetramethylbenzidinedihydrochloride (TMB) (0.7 μM)^42^ or fluorescent approaches based on carbon dots (0.45 μM)^43^ or boron nitride quantum dots (0.2 μM).^44^ Still, as the normal levels of GSH range from 500 to 15 000 μM, our strategy allows for the determination of such an analyte at physiological conditions.^45^

**Figure 4:**
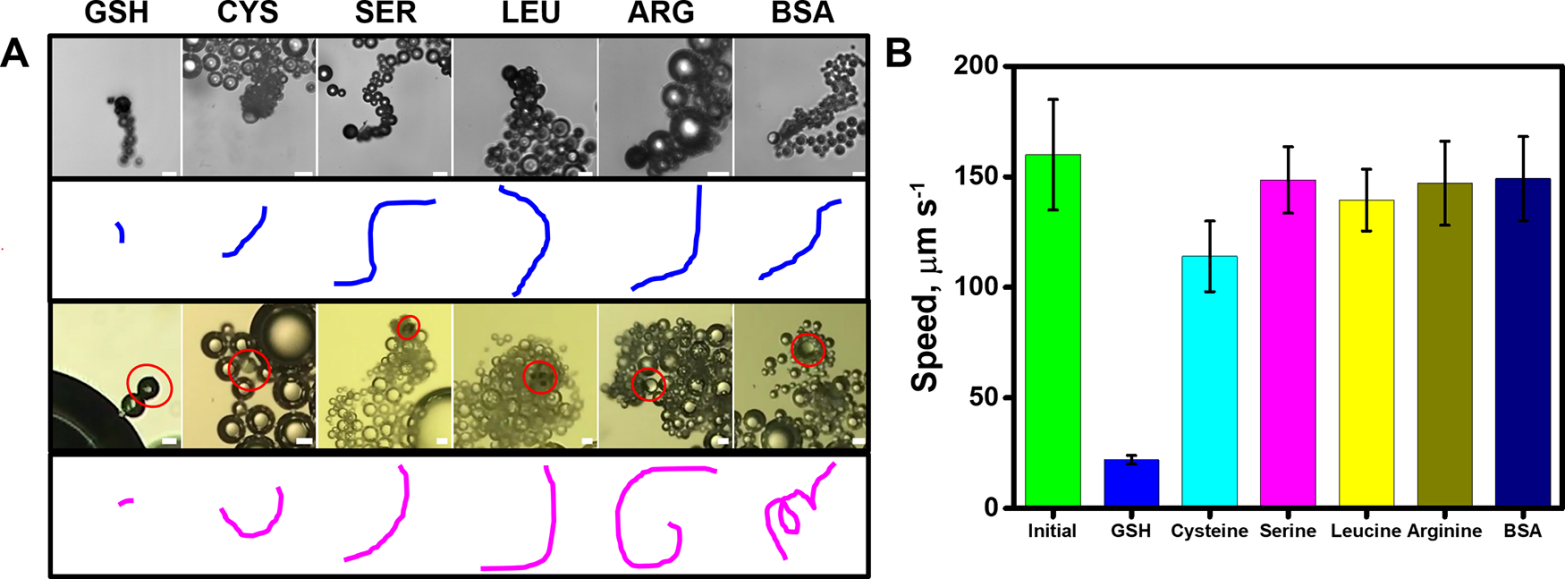
Selectivity of the detection strategy and detection performance. (A) Time-lapse images (taken from Video S4) and the corresponding tracking lines of micromotor navigation in solutions containing 160 μM of GSH, cysteine (Cys), serine (SER), leucine (LEU), arginine (ARG), and bovine serum albumin (BSA). The top part shows the images obtained with a smartphone and the bottom part the one obtained with a high-resolution optical microscope. (B) The corresponding speed in the presence of the different interferences. Scale bars, 20 μm.

Next, we evaluated the selectivity of the sensing strategy in the presence of other amino acids with a structure similar to that of GSH, which can cause potential interferences. Cysteine was tested because it contains a thiol group and an amino group in its structure. Serine, leucine, and arginine, which contain amino and COOH– groups, were also evaluated. In addition, bovine serum albumin, a protein commonly present in biological fluids and a well-known agent that can cause the poisoning of Pt catalyst, was also tested.^46^ As can be seen in the time-lapse images of Figure 4 and the corresponding Video S4, the speed of the micromotor does not change under the presence of 160 μM concentration of each interfering compound, as compared with the drastic decrease noted in the case of GSH. In the case of CYS, a thiol-containing compound, no decrease in the speed is observed up to a 100 μM concentration. At 160 μM, a slight decrease in the speed from 160 ± 25 to 114 ± 16 μm s^–1^ (*n* = 5 micromotors) is produced. For higher CYS concentrations (250–1000 μM), the speed decreases from 85 ± 5 μm s^–1^ to almost 0. Yet, much higher concentrations of cysteine (more than 250 μM) are needed to greatly affect the micromotor speed for quantitative purposes, whereas for GSH, only a 160 μM concentration is needed to completely stop the micromotors (for additional data, see Figure S1). This can be attributed to the relatively smaller molecular size of CYS (MW, 122 g mol^–1^) compared with GSH (MW, 307 g mol^–1^), thus higher concentrations of CYS are needed to completely block the Pt catalyst. In a previous work using tubular micromotors driven by Pt catalyst, a similar effect was reported for a 100 μM concentration of both compounds, where the initial micromotor speed decrease almost 2.6 times in the presence of GSH but 1.4 times in the presence of CYS.^27^ For BSA, a slight speed decrease to 149 ± 19 μm s^–1^ is noted, probably due to an increase in the viscosity of the samples. A marked decrease is produced by increasing the concentration up to 500 μM. Such results testified to the high selectivity of our sensing protocol. Selectivity was both evaluated with the smartphone and the optical microscope, obtaining similar results in both cases.

**Figure 5:**
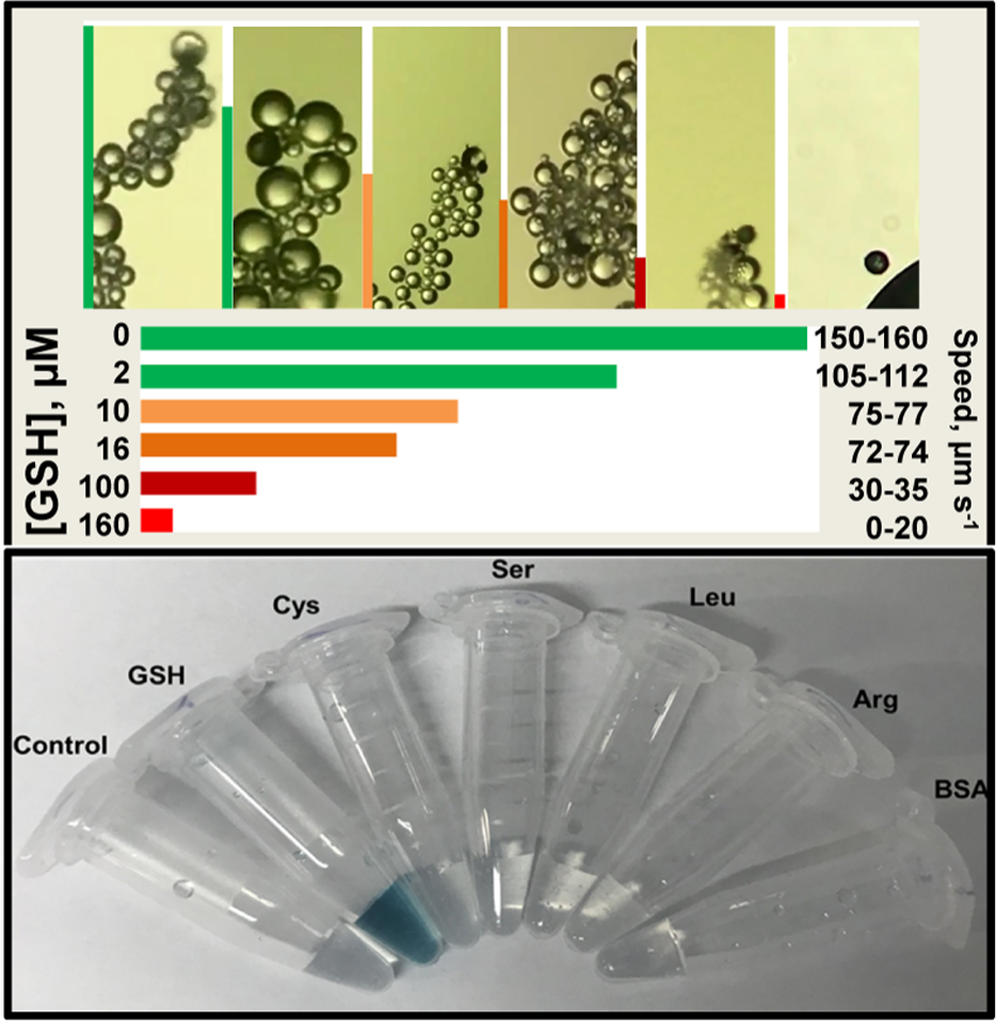
Protocol for GSH detection using the smartphone-based platform. Test strip readout based on the displacement of the micromotors in the real samples and correlation with GSH concentration and colorimetric evaluation under the presence of TMB as a substrate.

For future practical applicability by nonspecialized personnel, we developed a protocol for GSH sensing in clinical samples, as shown in Figure 5. As can be seen, the device can be prevalidated to construct the calibration plot in the presence of increasing concentrations of GSH. Pictures of the micromotor motion and displacement were taken, with a line at the left indicating the distance traveled by the micromotor. Differences in the bubble tail can be also checked. The strip correlates the distance traveled with a color and GSH concentration, so an operator just needs to observe the motion in the phone to make an estimate of GSH concentration for fast response. The detection can be performed in less than 30 s and just requires dropping the sample (human serum or plasma) on a glass slide along with the micromotor and fuel solution. The device can be used multiple times and the strategy can be extended to any smartphone as the magnifying lenses can be attached to any camera. The average micromotor cost per analysis is estimated to be 0.05€, considering the cost of peroxide, surfactant, and micromotors (in each synthesis, a total volume of 1 mL of micromotor suspension is obtained, but only 1 μL is required per analysis).

While effective and advantageous in terms of negligible sample processing, the developed smartphone micromotor-based platforms still have some limitations which prevent their true use as POC devices. First, low expeditiousness in sample processing (which requires dropping on the slide and individual measurement of the sample) needs to be addressed by the incorporation of automatic sampling in the future. Second, while direct observation of the sensor is possible, some training is required for micromotor focusing, monitoring, and speed tracking. This can be easily addressed by the development of specific software to be installed in a smartphone as an application, yet it can increase the cost. All this requires future works to transform the developed platform into a real POC device.

As a double check confirmation and complement to the motion-based test strip, a fast colorimetric test with the micromotors can be performed. To this end, TMB was used as a substrate. Under the presence of H_2_O_2_ and nanomaterials with peroxidase-like activity (graphene, platinum nanoparticles), OH^–^ radicals are oxidized, generating a blue color in the solution.^47,48^ Surprisingly, under the presence of the interferences, no blue color was developed, probably because all of the peroxide was decomposed into oxygen and water without the generation of intermediate OH^–^ radicals by the high catalytic activity of PtNPs.^49^ Yet, under the presence of GSH, the deep blue color is generated. Although some authors have reported that GSH can reduce back TMB, resulting in a decrease in color,^50,51^ we observed the opposite. Color appearance in the solution is observed at concentrations up to 10 μM of GSH. We hypothesize that all GSH attaches to the PtNPs in the micromotors, thus blocking the active part responsible for the reduction of GSH. This, in turn, reduces the catalytic activity, thus some H_2_O_2_ in the solution can react with the graphene oxide present in the micromotor, generating OH^–^ ions that oxidize the TMB and generate the blue color. We tested also the colorimetric TMB assay in the presence of the 1000 μM concentration of CYS. No blue color is generated, probably because CYS at a high concentration can inhibit TMB oxidation to generate a blue product, as described in the previous report.^52^

**Figure 6:**
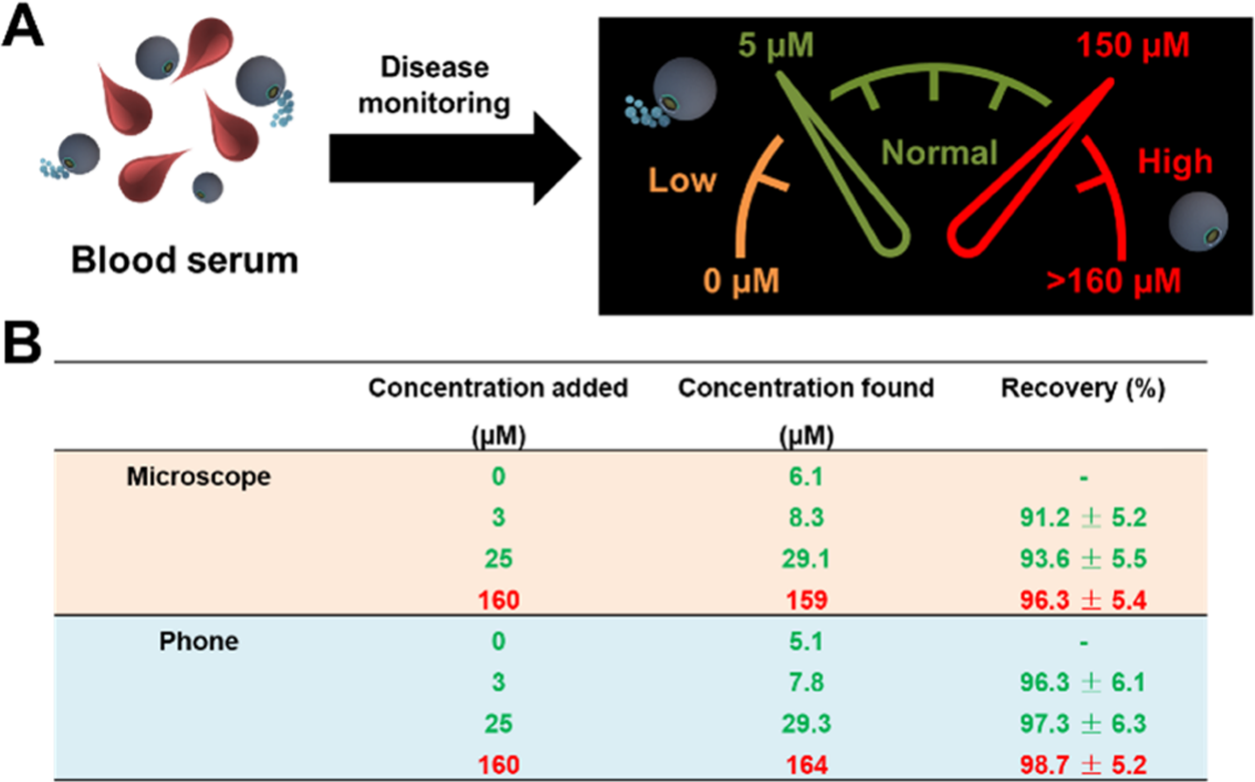
Health and disease state prognosis using the micromotor-based POC device for GSH detection. (A) Schematic illustration of the concept consisting of blood serum dilution, measurement, and corresponding GSH levels related to health and potential nonhealthy conditions. (B) GSH determination in diluted human serum and recoveries obtained with a microscope and a smartphone (*n* = 3).

To illustrate the applicability of the smartphone-based platform, we fortified human serum samples with different levels of GSH to simulate a protocol for further application in disease monitoring. It is well-known that GSH levels in normal serum samples are within the range from 500 to 15 000 μM.^53^ Thus, to fit the linear range reported for our method, we diluted the samples nearly 100-fold with the PBS buffer, thus GSH is within the range from 5 to 150 μM after dilution. Before performing recovery experiments, we applied our strategy (both using the microscope and the phone device) for the detection of GSH in the serum samples (100-fold dilution) prior to fortification. As can be seen in Figure 6, the measured concentration of GSH in serum is about 6 μM (600 mM), which is within the normal reported range. Such a figure also shows an example of a schematic of disease monitoring using our device, with an associated concentration range of “gluthationemeter” (considering the 100-fold dilution of the serum sample). Low levels of GSH (bellow 5 μM, in orange in Figure 6A) are indicative of the oxidative stress associated with Alzheimer’s and Parkinson’s diseases, as well as health failure or diabetes.^54,55^ Values from 5 to 150 μM (in green in Figure 6A) indicate normal GSH levels. High GSH levels (in red in Figure 6A) have been associated with cancer and can induce tumor resistance to chemotherapy.^56,57^ The micromotor-based smartphone device reported here can be used to monitor the level of GSH in cancer patients along with other biomarkers, with high levels associated with the tumor state and efficiency of the treatment.

Additional recovery experiments also testified to the feasibility of the smartphone platform for disease monitoring. We assayed concentration at all of the range studies (low–normal–high), obtaining excellent recoveries in all cases, ranging from 91.2 to 98.7%. Such values indicate quantitative analyte measurement, assuring the validity of the analytical method for its application in real samples.

## Conclusions

We have reported here a smartphone portable device based on micromotors for the naked-eye detection of clinical relevance biomarkers for future decentralized analysis and use in remote settings. The coupling of a high-magnification lens allows to directly observe and record videos of the motion of 20 μm graphene-wrapped/PtNP Janus micromotors using a phone camera in an easy manner. Yet, while Janus micromotors are used here to illustrate the concept, the strategy can be easily extended to other designs such as wires, tubular, etc. The concept was successfully illustrated for the motion-based detection of glutathione, an analyte of high clinical relevance, with excellent selectivity and practical levels. A test strip that correlates the distance traveled with GSH concentration allows for direct observation of the micromotor motion in the phone to make an estimate of GSH concentration for fast response. The detection can be performed in less than 30 s and just requires dropping the sample (human serum or plasma) on a glass slide along with the micromotor and fuel solution, with an average cost of 0.05€ per analysis. The validation against a high-performance optical microscope further testifies the utility of our universal device. As such, the smartphone device meets the World Health Organization’s criteria (affordable, sensitive, specific, user-friendly, rapid, equipment-free). Future efforts should be aimed at extending the strategy to other micromotor-based sensing approaches relying on fluorescence or colorimetric detection for the design of multiplexed schemes. Current challenges such as low expeditiousness and the development of specific software to simplify speed measurement still need to be addressed.

## Supporting Information Available

Selectivity of the detection strategy in the presence of high concentrations of CYS and BSA (Figure S1) (PDF)
Smartphone device assembly (Video S1) (AVI)
Janus micromotor navigation in the absence and presence of glutathione using the smartphone-based platform (Video S2) (AVI)
Janus micromotor navigation in solutions containing increasing concentrations of GSH, using a high-resolution optical microscope and the smartphone-based platform (Video S3) (AVI)
Selectivity of the strategy using a high-resolution optical microscope and the smartphone-based platform (Video S4) (AVI)

## Supplementary Material

ac1c02947_si_001.pdf

ac1c02947_si_002.avi

ac1c02947_si_003.avi

ac1c02947_si_004.avi

ac1c02947_si_005.avi
