# Supplementary material for: Smartphone-Based Janus Micromotors Strategy for Motion-Based Detection of Glutathione
Source: Anal Chem. 2021 Nov 22;93(49):16385–92. doi: 10.1021/acs.analchem.1c02947 (PMC8674879; doi:10.1021/acs.analchem.1c02947)
Supplement: Supplementary file 1 — ac1c02947_si_001.pdf [file ac1c02947_si_001.pdf]

Supporting information

Smartphone-based Janus micromotors strategy for motion-based detection of glutathione

Kaisong Yuan,[†#] Carmen Cuntín-Abal,[†] Beatriz Jurado-Sánchez,[†‡*] and Alberto Escarpa[†‡ *]

[†]Department of Analytical Chemistry, Physical Chemistry and Chemical Engineering, University of Alcala, Alcala de Henares E-28871, Madrid, Spain. E-mail: beatriz.jurado@uah.es, alberto.escarpa@uah.es (Tel: +34 91 8854995)

[#]Shantou University Medical College, No. 22, Xinling Road, Shantou 515041, China

[‡]Chemical Research Institute "Andrés M. del Río", University of Alcala, Alcala de Henares E-28871, Madrid, Spain

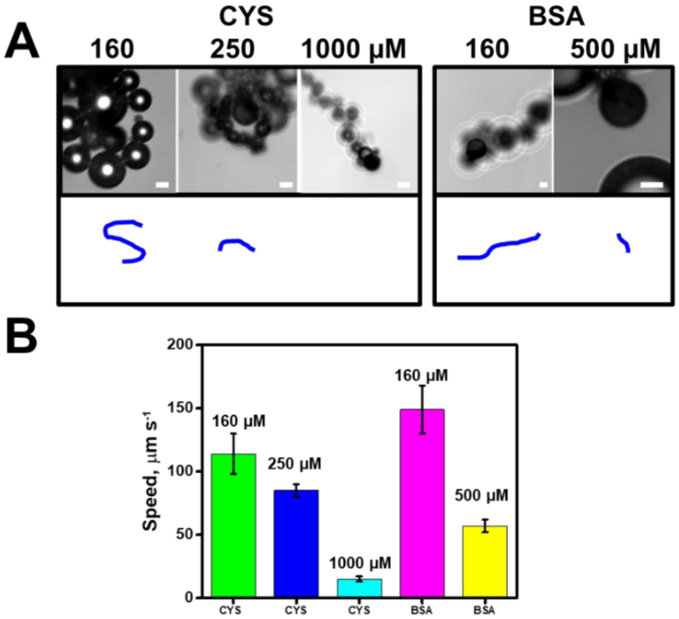

Figure S1. Selectivity of the detection strategy in the presence of high concentrations of CYS and BSA. A) Time-lapse images and corresponding tracking lines of micromotors navigation in solutions containing CYS and BSA. B) Corresponding speed in the presence of the different concentration interferences. Scale bars, 10 µm. Please note that the concentrations assayed are higher than the normally present in human blood, which ranged from 240-360 µM in the case of CYS[1] and 530 to 750 µM in the case of human serum albumin as analogous to BSA[2].

1. Rehman T., Shabbir M.A., Inam-Ur-Raheem M., Manzoor M.F., Ahmad N.H., Siddeeg A., Abid M., Aadil R.M., Cysteine and homocysteine as biomarker of various diseases, Food Sci. Nutr., **2020**, *8*, 4696-4707.

2. Mishra V., Heath R.J., Structural and Biochemical Features of Human Serum Albumin Essential for Eukaryotic Cell Culture, Int. J. Mol. Sci., **2021**, *22*, 8411
